# Body Composition May Be a Novel Presurgical Risk Factor for Acute Kidney Injury among Clear Cell Renal Cell Cancer Patients Undergoing Radical Nephrectomy

**DOI:** 10.15586/jkc.v12i4.423

**Published:** 2025-10-17

**Authors:** Linnea T. Olsson, Alejandro Sanchez, Marina Mourtzakis, A. Ari Hakimi, Paul Russo, Edgar A. Jaimes, Patrick T. Bradshaw, Helena Furberg

**Affiliations:** 1 Department of Health Behavior, Gillings School of Global Public Health, University of North Carolina at Chapel Hill, Chapel Hill, NC;; 2 Department of Epidemiology, Gillings School of Global Public Health, University of North Carolina at Chapel Hill, Chapel Hill, NC;; 3 Department of Surgery, Division of Urology, Huntsman Cancer Institute, University of Utah, Salt Lake City, Utah;; 4 Department of Kinesiology, University of Waterloo, Waterloo, Canada;; 5 Department of Surgery, Division of Urology, Memorial Sloan Kettering Cancer Center, New York, NY;; 6 Renal Service, Memorial Sloan Kettering Cancer Center, New York, NY;; 7 Division of Epidemiology, School of Public Health, University of California Berkeley, Berkeley, CA, USA;; 8 Department of Epidemiology and Biostatistics, Memorial Sloan Kettering Cancer Center, New York, NY

**Keywords:** acute kidney injury, body composition, kidney cancer, kidney function, muscle, visceral adipose tissue

## Abstract

Patients with renal cell carcinoma (RCC) undergoing nephrectomy are at risk for acute kidney injury (AKI). Prior studies have focused predominantly on nonmodifiable surgical AKI risk factors. We conducted the first investigation of body composition features and AKI to identify factors that could improve presurgical risk stratification and be targeted in future interventions. We analyzed data from 1199 patients with stages I–III, clear cell RCC undergoing radical (RN) or partial nephrectomy (PN) from 2000 to 2020. AKI was defined as a serum creatinine (sCr) increase by 0.3 mg/dL within 48 h or a 1.5-fold increase in sCr within 7 days. Preoperative computed tomography (CT) scans were segmented to determine quantities and radiodensities of adipose tissue and skeletal muscle using Automatica software. Multivariable generalized linear models estimated 7-day risk differences (RD) and 95% confidence intervals (CI) within surgical subgroups. AKI was more frequent among patients undergoing RN (66%) than PN (26%). For RN, only higher visceral adipose tissue (VAT) quantity was significantly associated with greater AKI risk (RD per 40-unit increase 5.2 [95% CI: 1.3, 9.2]). We initially detected a similar association in PN, but after multivariable adjustment for all body composition features, associations were attenuated and became nonsignificant. Associations between presurgical body composition and AKI risk vary by surgery type. Higher VAT quantity increased AKI risk only among RN patients. If confirmed, CT-derived VAT quantity may be a novel presurgical imaging characteristic that could be used to inform treatment selection or modified to decrease postoperative AKI risk in RN patients.

## Introduction

Among renal cell carcinoma (RCC) patients, postoperative acute kidney injury (AKI) is common after nephrectomy and increases the long-term risk of chronic kidney disease (CKD) (1, 2). AKI incidence varies widely based on surgery type, with reported rates ranging from 13–47% for radical nephrectomy (RN) and 9–41% for partial nephrectomy (PN) (3). Prior studies examining risk factors for postoperative AKI have primarily evaluated nonmodifiable risk factors (e.g., age, tumor size) and surgical factors such as the amount of normal parenchymal mass preserved (4–6). Identifying new modifiable factors for AKI could help identify patients at higher risk for AKI and inform the development and testing of presurgical interventions.

Higher body mass index (BMI) is an established risk factor for developing RCC and CKD (7, 8). The few studies that examined the impact of presurgical BMI on AKI risk in RCC are inconclusive or contradictory (2, 9, 10). This is likely because BMI does not distinguish between tissue types, which can exert distinct biological effects (11). Both skeletal muscle and visceral adipose tissue features are emerging as novel prognostic factors in RCC (12, 13). Presurgical computed tomography (CT) scans can be used to measure the quantity (cross-sectional area) and quality (radiodensity) of skeletal muscle, and visceral and subcutaneous adipose tissue. Host-level visceral adipose tissue (VAT) characteristics (both the quantity and quality) can be measured from CT scans, but, to our knowledge, have never been evaluated in relation to AKI risk in RCC (14).

To address this gap, we analyzed presurgical CT-derived body composition features in relation to AKI risk in a well-characterized cohort of localized clear cell RCC (ccRCC) patients who underwent radical or partial nephrectomy (RN or PN, respectively). We hypothesized that having more visceral fat (increased VAT) would be associated with an increased risk of AKI regardless of surgery type because of the heightened metabolic demands of excess adiposity imposed on the normal remaining kidney(s) (15).

## Materials and Methods

### Patient population

The RESOLVE study is a retrospective cohort study approved by the Memorial Sloan Kettering (MSK) Institutional Review Board. The study design has been previously described (16). The study included 1239 patients aged 18–85 who underwent either RN or PN at MSK for nonmetastatic (stages I–III) ccRCC from 2000 to 2020. To ensure that preoperative CT scans accurately reflect body composition at the time of surgery, we only included patients with a CT scan taken within 3 months before nephrectomy (12). The eligible patients had no history of cancer or perioperative systemic therapy. For this analysis, we excluded patients who had a solitary kidney (n = 2), had missing preoperative serum creatinine (sCr; n = 2), or did not have at least one sCr value within 7 days post-surgery (n = 36), resulting in a final analytic cohort of 1199 ccRCC patients. The median time from preoperative sCr measurement to surgery was 8 days (IQR 0–62 days).

### Outcome assessment

Patients were classified as having AKI based on the KDIGO classification, defined as an increase in (a) absolute sCr by 0.3 mg/dL within 48 h or (b) 1.5 times sCr increase from baseline within 7 days (17).

### Clinicodemographic data

The following information was available for each patient from a prospectively maintained clinical database at MSK: demographics (age at surgery, biological sex, and race), smoking history (ever vs never), and comorbidities (lifetime history of and/or treatment for diabetes, hypertension, and/or hyperlipidemia). Height (m) and weight (kg) were measured during the presurgical visit and used to calculate BMI (kg/m^2^), which was analyzed as a continuous variable. Perioperative urine albumin/creatinine ratio measurements were not available for abstraction as they are not routinely measured in this patient population.

### Operative details

Surgery-related variables were abstracted from the electronic medical record, including surgery type (RN vs PN) and approach (open vs laparoscopic/robotic). Ischemia type (warm vs cold) and ischemic time (in minutes) were documented for PN patients only. Based on the prior literature (5), ischemic times of ≥ 25 min for warm ischemia (laparoscopic/robotic approach) or ≥ 40 min for cold ischemia (open approach) were regarded as “prolonged.” The estimated glomerular filtration rate (eGFR) was calculated using the CKD-EPI Creatinine Equation (2021) (18). Baseline chronic kidney disease (CKD; yes/no) was defined as eGFR ≤ 60 mL/min (CKD stage 3a or higher) (19).

### Pathologic details

Tumor characteristics were abstracted from pathology reports and included tumor size (cm), tumor stage (pT), lymph node involvement (pN), AJCC stages (I–III), and histologic grade (I–IV).

### Body composition assessment

**Figure S1:**
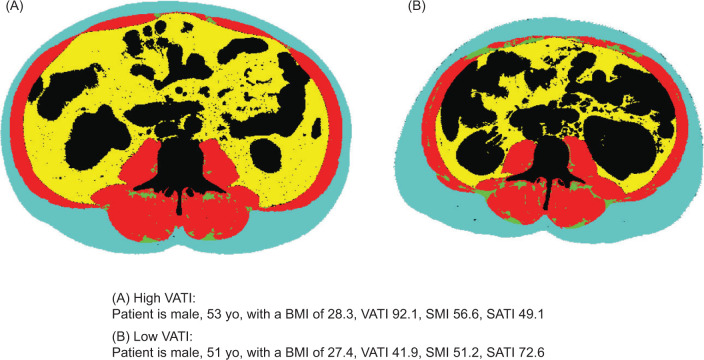
Images of segmented CTs from two patients in Resolve representing high (A) and low (B) VATI. While both patients have similar BMIs, patient A has approximately 50 cm^2^/m^2^ higher VATI (segmented in yellow) than patient B. SMI is represented in red, while SATI is represented in cyan.

The BMI poorly reflects body composition. Methods to quantify body composition features in our cohort have been previously described (16). Skeletal muscle (psoas, erector spinae, quadratus lumborum, transverse abdominis, rectus abdominis, as well as internal and external obliques) and adipose tissue (visceral or subcutaneous) depots were measured on a single CT image at the L3 vertebrae using Automatica software (20). Established Hounsfield unit (HU) threshold values were used to segment the CT images into distinct tissue types. Segmentation resulted in continuous cross-sectional area values (CSA; cm^2^) and radiodensity (HU) for each tissue type (21). Index measurements for each tissue type were calculated by dividing CSAs by height (m) squared. We evaluated how the index (tissue quantity) and radiodensities (tissue quality) of skeletal muscle, and visceral and subcutaneous adiposity were associated with AKI risk (i.e., SMI, SMD, VATI, VATD, SATI, and SATD). *Lower* SMD, reflecting fat-infiltrated muscle, but *higher* VATD and SATD, reflecting fat-depleted adipocytes, were considered pathological (22–24). Supplemental Figure 1 shows the differences in body composition features for two male patients with similar BMI.

**Figure 1:**
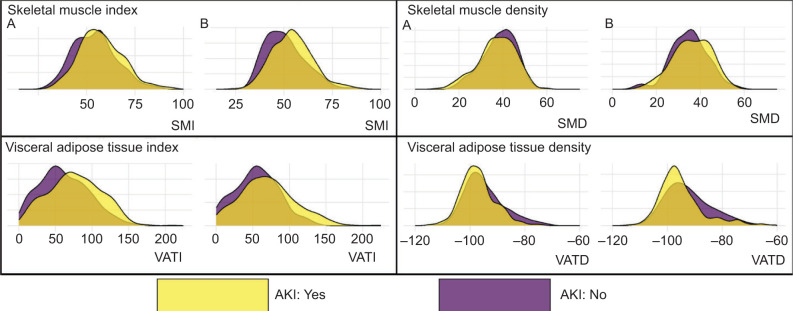
Density plots showing the distribution of each body composition variable (skeletal muscle index – SMI, skeletal muscle density – SMD, visceral adipose tissue index – VATI, visceral adipose density – VATD) in either (A) partial nephrectomy or (B) radical nephrectomy cohort. Density plots are stratified by AKI (yes is gold, no is purple).

### Statistical analysis

All statistical analyses were performed with R (version 4.3.2). Given the known differences in short and long-term kidney function outcomes after PN versus RN, we present surgery-stratified analyses (1, 25). We used generalized linear models with a binomial family and identity link to estimate 7-day risk differences (RD) and 95% confidence intervals (26). RDs are the absolute change in risk of experiencing the outcome per unit increase in body composition variable, holding any other variables in the model constant. We scaled body composition variables so that a unit of change corresponded to approximately one standard deviation (SD) change based on the distribution of each body composition measure in the overall population. This scaling was chosen to yield a consistent and meaningful magnitude of change in each exposure across the models considered. For SMI, SMD, SATD, and VATD, this translated to a 10-unit (cm^2^/m^2^ for SMI, HU for SMD/VATD) change in the continuous variable, while for VATI and SATI, we scaled to a 40 cm^2^/m^2^ change because of their wider range of values. We present univariate estimates for individual body composition variables and age-, sex-, and comorbidity-adjusted estimates for multivariable models with individual and simultaneous inclusion of the body composition variables. Although perioperative factors, particularly ischemia time for PN, have been associated with AKI risk, we did not include this feature in multivariable models because it is an intermediate between our exposure (body composition measurement) and outcome (AKI). Therefore, ischemia time does not meet the criteria for confounding (27). All statistical tests used a significance level of 0.05.

## Results

### Radical nephrectomy

Acute kidney injury occurred in 66% of patients undergoing RN (Table 1). Patients who underwent RN (Table 1) had a median age of 60 years, a median BMI of approximately 30 kg/m^2^, and 70% were males. Patients had close to normal kidney function at baseline (median eGFR 76 mL/min; IQR 64, 92), and 83% had a preoperative eGFR over 60 mL/min. Most RN patients had higher-stage disease (61% stage 3 tumors).

Patients who underwent RN and developed AKI were more likely to be males, had a history of hyperlipidemia, and had a higher median baseline eGFR relative to those who did not; correspondingly, patients who experienced AKI were less likely to be classified as having CKD at baseline. Patients with AKI had lower-stage, smaller tumors and were more likely to have received an open surgery.

**Table 1:** Demographic and clinical characteristics of the patient cohort, restricted to those receiving radical nephrectomy (n = 446), stratified by acute kidney injury (AKI) in the seven days following nephrectomy.

Variable	Overall, N = 446 (100%)	No AKI, N = 153 (34.3%)	AKI, N = 293 (65.7%)
Age (yrs), median (IQR)	60 (52, 67)	60 (54, 66)	60 (51, 67)
Sex			
Female	135 (30%)	73 (48%)	62 (21%)
Male	311 (70%)	80 (52%)	231 (79%)
Race			
White	393 (88%)	135 (88%)	258 (88%)
Asian	22 (4.9%)	8 (5.2%)	14 (4.8%)
Black	15 (3.4%)	5 (3.3%)	10 (3.4%)
Other	7 (1.6%)	2 (1.3%)	5 (1.7%)
Unknown	9 (2.0%)	3 (2.0%)	6 (2.0%)
eGFR at baseline (mL/min/1.73 m2)	76 (64, 92)	72 (61, 84)	79 (67, 94)
CKD (eGFR < 60)			
Yes	78 (17%)	36 (24%)	42 (14%)
No	368 (83%)	117 (76%)	251 (86%)
Stage at diagnosis			
1	125 (28%)	40 (26%)	85 (29%)
2	48 (11%)	10 (6.5%)	38 (13%)
3	273 (61%)	103 (67%)	170 (58%)
Tumor size (cm), Median (IQR)	6.7 (4.6, 9.0)	7.4 (5.0, 9.8)	6.4 (4.5, 8.5)
Surgical approach			
Laparoscopic/Robotic	84 (19%)	37 (24%)	47 (16%)
Open	362 (81%)	116 (76%)	246 (84%)
BMI (kg/m2), median (IQR)	29.6 (26.4, 33.5)	30.0 (25.8, 33.7)	29.4 (26.6, 33.5)
History of diabetes	77 (17%)	29 (19%)	48 (16%)
History of hypertension	272 (61%)	96 (63%)	176 (60%)
History of hyperlipidemia	163 (37%)	48 (31%)	115 (39%)

AKI = acute kidney injury. BMI = body mass index, CKD = chronic kidney disease, eGFR = estimated glomerular filtration rate.

**Table 2:** Demographic and clinical characteristics of the patient cohort, restricted to those receiving partial nephrectomy (n = 754), stratified by acute kidney injury (AKI) in the seven days following nephrectomy.

Variable	Overall, N = 754 (100%)	No AKI, N = 559 (74.1%)	AKI, N = 195 (25.9%)
Age (continuous)	57 (48, 64)	56 (48, 64)	58 (48, 64)
Sex			
Female	242 (32%)	203 (36%)	39 (20%)
Male	512 (68%)	356 (64%)	156 (80%)
Race			
White	674 (89%)	496 (89%)	178 (91%)
Asian	32 (4.2%)	28 (5.0%)	4 (2.1%)
Black	19 (2.5%)	10 (1.8%)	9 (4.6%)
Other	10 (1.3%)	10 (1.8%)	0 (0%)
Unknown	19 (2.5%)	15 (2.7%)	4 (2.1%)
eGFR at baseline	81 (68, 97)	80 (69, 97)	83 (68, 97)
CKD (eGFR < 60)			
Yes	87 (12%)	65 (12%)	22 (11%)
No	667 (88%)	460 (88%)	207 (89%)
Stage at dx			
1	649 (86%)	495 (89%)	154 (79%)
2	12 (1.6%)	7 (1.3%)	5 (2.6%)
3	93 (12%)	57 (10%)	36 (18%)
Tumor size	3.00 (2.1, 4.2)	2.90 (2.0, 4.0)	3.50 (2.5, 4.5)
Surgical approach			
Laparoscopic/Robotic	212 (28%)	154 (28%)	58 (30%)
Open	542 (72%)	405 (72%)	137 (70%)
Ischemic time#	29 (21, 40)	28 (20,37)	35 (25,45)
Prolonged ischemic time*			
Yes	230 (33%)	140 (27%)	90 (51%)
No	469 (67%)	381 (73%)	88 (49%)
Missing	55	38	17
BMI	29.6 (26.5, 33.4)	29.3 (26.3, 32.8)	30.5 (27.4, 34.3)
History of diabetes	109 (14%)	75 (13%)	34 (17%)
History of hypertension	380 (50%)	270 (48%)	110 (56%)
History of hyperlipidemia	294 (39%)	213 (38%)	81 (42%)

* Based on the prior literature (5), ischemic times of ≥ 25 min for warm ischemia (laparoscopic/robotic approach) or ≥ 40 min for cold ischemia (open approach) were regarded as “prolonged.”

# Excluded 55 overall without ischemia time (no AKI = 28, AKI = 17).

AKI: Acute kidney injury; BMI: Body mass index; CKD: Chronic kidney disease; eGFR: Estimated glomerular filtration rate.

### Partial nephrectomy

Acute kidney injury occurred in 26% of patients undergoing PN (Table 2). Patients undergoing PN (Table 2) had a median age of 57 years, a median BMI of approximately 30 kg/m^2^, and 68% were males. Overall, patients had normal kidney function at baseline (median eGFR 81 mL/min; IQR 68, 97), and 88% had a preoperative eGFR above 60 mL/min. Most patients undergoing PN had stage I disease (86%). An open surgical approach was most common among these patients (72%), and approximately one-third of surgeries were classified as having prolonged ischemic time.

Male patients had a higher frequency of AKI than female patients in both RN and PN groups. Among patients who underwent PN, those with AKI had slightly more comorbidities and presented with higher-stage and larger tumors than those without AKI. Patients who experienced AKI had a substantially higher frequency of prolonged ischemic time than those who did not.

### Body composition and AKI risk in the RN cohort

The overall distribution of body composition variables in the RN cohort is shown in Figure 1A. Among the RN cohort (Table 3), in univariate unadjusted models, lower SMI, lower SMD, and higher VATD were initially associated with a lower AKI risk, while higher VATI was associated with an increased AKI risk. However, after adjustment for age, sex, and comorbidities, only lower SMI and higher VATI remained significantly related to AKI risk. The final multivariable model accounted for all body composition variables and revealed that only higher VATI (per 40-unit increase) was significantly associated with AKI risk in RN patients (Table 3; RD: 5.2%, 95% CI: 1.3, 9.2). Distributions of clinicopathological variables by VATI are shown in Supplemental Table 1.

**Table 3:** Analytic models estimating associations between body composition and risk of AKI among patients who received radical nephrectomy (RN, n=446). The absolute risk of incident AKI among all patients receiving RN was 65.7%.

	Model 1: Individual Features RD (95% CI)	Model 2: Individual Features with age, sex, and comorbidities1 RD (95% CI)	Model 3: Fully-adjusted with body comp variables, age, sex, and comorbidities2 RD (95% CI)
SMI (per 10-unit decrease)	–8.8 (–12.2, –5.3)	–5.2 (–10.3, –0.1)	–3.5 (–9.3, 2.2)
SMD (per 10 HU decrease)	–5.8 (–11.5, –0.2)	–2.1 (–8.3, 4.2)	–1.7 (–9.5, 6.0)
VATI (per 40-unit increase)	8.8 (7.5, 10.1)	6.4 (4.1, 8.8)	5.2 (1.3, 9.2)
VATD (per 10 HU increase)	–5.6 (–11.8, 0.6)	–4.7 (–10.5, 1.1)	–1.6 (–11.0, 7.7)
SATI (per 40-unit increase)	–6.1 (–10.4, –1.8)	–2.5 (–7.2. 2.1)	–5.3 (–10.9, 0.2)
SATD (per 10 HU increase)	1.0 (–4.9, 6.9)	–1.6 (–7.4, 4.2)	–1.7 (–10.6, 7.3)

Comorbidities include histories of hypertension, diabetes, or hyperlipidemia.

Body composition variables include SMI, SMD, VATI, VATD, as well as SATI (subcutaneous adipose tissue index) and SATD (subcutaneous adipose tissue density).

### Body composition and AKI risk in the PN cohort

The overall distribution of body composition variables in the PN cohort is shown in Figure 1B. Among the PN cohort (Table 4), only higher VATI was initially associated with increased AKI risk. However, after multivariable adjustment for all body composition features, associations were attenuated and became nonsignificant.

**Table 4:** Analytic models estimating associations between body composition and risk of AKI among patients who received partial nephrectomy (PN, n=754). The absolute risk of incident AKI among all patients receiving PN was 25.9%.

	Model 1: Individual Body Composition Features RD (95% CI)	Model 2: Individual Body Composition Features with age, sex, and comorbidities1 RD (95% CI)	Model 3: Fully-adjusted with all Body Composition Features, age, sex, and comorbidities2 RD (95% CI)
SMI (per 10-unit decrease)	–5.7 (–8.6, –2.9)	–4.6 (–8.3, –0.1)	–3.7 (–7.6, 0.2)
SMD (per 10 HU decrease)	1.1 (–3.2, 5.4)	2.7 (–1.9, 7.4)	2.4 (–2.1, 6.9)
VATI (per 40-unit increase)	9.1 (5.4, 12.9)	6.5 (1.9, 11.1)	3.6 (–3.0, 10.2)
VATD (per 10 HU increase)	–8.2 (–11.8, –4.6)	–5.2 (–9.0, –1.4)	–3.5 (–9.7, 2.7)
SATI (per 40-unit increase)	–0.1 (–3.0, 2.8)	1.7 (–1.2, 4.6)	–1.7 (–4.9, 1.5)
SATD (per 10 HU increase)	–0.5 (–5.8, 4.7)	–2.0 (–6.7, 2.8)	1.1 (–4.9, 7.0)

Comorbidities include histories of hypertension, diabetes, or hyperlipidemia.

Body composition variables include SMI, SMD, VATI, VATD, as well as SATI (subcutaneous adipose tissue index) and SATD (subcutaneous adipose tissue density).

## Discussion

To our knowledge, this is the first investigation of presurgical body composition features and AKI risk in localized ccRCC. We found that associations between body composition and risk of AKI differ by surgery type, such that higher VATI was associated with increased AKI risk only among patients undergoing RN. Body composition features were not significantly associated with AKI risk among patients undergoing PN after mutual adjustment for all body composition features. Our findings, if validated, suggest that CT-derived body composition segmentation may be a novel way to identify those at greater risk of AKI before RN. In addition, since body composition is modifiable, future studies could evaluate whether decreasing VATI through presurgical behavioral, pharmacologic, or surgical interventions ameliorates AKI risk in patients undergoing RN.

Our results align with prior research conducted among the general and noncancer populations (i.e., diabetics) that directly measured adipose tissue features from imaging and reported that increasing perirenal adipose tissue thickness is associated with worse kidney function (16, 28–30). Notably, perirenal adipose tissue is a component of VAT but only represents a subset of a patient’s total VAT quantity. Our findings suggest that total VAT quantity is a risk factor for AKI only among ccRCC patients undergoing RN. We found no associations between AKI risk and SAT quantity or quality. This supports the idea that VAT is considered more biologically active than SAT and is associated with metabolic dysfunction and heightened systemic inflammation (31).

The distinct influence of VATI on AKI risk only in RN may be explained by the one-kidney versus two-kidney model of renal function. Body composition plays a limited role in AKI risk in PN (two-kidney model) because the percent of preserved normal renal parenchyma during PN is the most critical factor influencing short- and long-term renal function (5, 6, 9). In PN, renal parenchymal preservation is influenced by surgical characteristics, including the approach to the renal mass (enucleation vs wide resection), cold versus warm ischemia, and the renorrhaphy technique (single vs double layer) (32). In contrast, patients undergoing RN (one-kidney model) have less preserved normal parenchyma, and the remaining contralateral kidney must compensate for the lost functional tissue and respond to perioperative renal insults (e.g., low blood pressure). Therefore, the metabolic dysfunction and inflammation caused by higher VATI could result in more injury after RN, since the single-kidney model may not handle these stressors as well as the two-kidney model.

Our study has several strengths, including the large sample size of well-characterized patients and multivariable analyses that accounted for both the quantity and quality of all body composition variables. As demonstrated by our prior publications, controlling for all body composition features simultaneously in multivariable models is essential to identify which tissue type is associated with the outcome of interest (13, 16). We acknowledge study limitations. First, our body composition segmentation program does not specifically measure perirenal fat thickness, which precluded our ability to determine the correlation between perirenal adipose thickness and VATI. Therefore, we cannot determine if they represent the same exposure or contribute to separate localized versus systemic effects. Second, as skeletal muscle is an essential source of creatinine, which was used to determine eGFR, it is possible that kidney function values were inflated for patients with higher skeletal muscle quantity (3). To address this possibility, we accounted for a patient’s quantity and quality of skeletal muscle in multivariable models. Future studies could use a non-muscle-dependent measure such as cystatin C, which may more reliably estimate renal function in patients outside of “normal” body phenotype. Finally, we could not access medications that may influence renal function (e.g., angiotensin receptor blockers). However, these are routinely stopped before surgery as a part of the standard of care and, therefore, unlikely to influence AKI risk significantly.

The future of this work could involve assessing preoperative body composition features from existing CT scans as a new “vital sign” to estimate short-term renal function outcomes, influence decisions between RN versus PN, and design lifestyle/pharmacologic interventions to reduce VATI. As a modifiable risk factor, VAT quantity can be modified safely between renal mass diagnosis and surgery and in the postoperative period (33). For example, GLP-1 agonists could be tested to reduce the quantity of visceral adipose tissue and improve long-term renal and cardiovascular outcomes (34).

## Conclusions

Identifying modifiable risk factors for AKI may reduce CKD risk after nephrectomy, and provide targeted clinical management and counseling opportunities (35, 36). Our large-scale study suggests that ccRCC patients with higher VATI at the time of RN are at higher risk of developing AKI. VATI represents a novel imaging characteristic that may identify localized ccRCC patients at the highest risk of postoperative AKI. If our findings are confirmed in other studies, presurgical interventions to modify body composition could be tested to reduce AKI risk in patients undergoing RN.

## Figures and Tables

**Table S1:** Patient and tumor characteristics, stratified by VATI (dichotomized as low/high based on sex-specific medians).

Characteristic	Overall N = 445 *	Low VATIN = 224* (50.3%)	High VATIN = 221* (59.7%)
Age	60 (52, 67)	58 (49, 65)	62 (55, 68)
Sex			
Female	134 (30%)	57 (25%)	77 (35%)
Male	311 (70%)	167 (75%)	144 (65%)
Race			
Asian	22 (4.9%)	14 (6.3%)	8 (3.6%)
Black	15 (3.4%)	11 (4.9%)	4 (1.8%)
Other	7 (1.6%)	4 (1.8%)	3 (1.4%)
Unknown	9 (2.0%)	3 (1.3%)	6 (2.7%)
White	392 (88%)	192 (86%)	200 (90%)
eGFR at baseline	76 (64, 92)	78 (66, 93)	74 (62, 90)
CKD (eGFR < 60)			
0	344 (77%)	182 (81%)	162 (73%)
1	101 (23%)	42 (19%)	59 (27%)
Stage at dx			
1	125 (28%)	58 (26%)	67 (30%)
2	48 (11%)	29 (13%)	19 (8.6%)
3	272 (61%)	137 (61%)	135 (61%)
Tumor size	6.60 (4.60, 9.00)	7.25 (4.85, 9.80)	6.40 (4.50, 8.30)
Surgical approach			
Laparoscopic/Robotic	84 (19%)	43 (19%)	41 (19%)
Open	361 (81%)	181 (81%)	180 (81%)
BMI	29.6 (26.4, 33.5)	26.9 (24.3, 30.1)	32.7 (29.2, 37.6)
History of diabetes	76 (17%)	16 (7.1%)	60 (27%)
History of hypertension	271 (61%)	103 (46%)	168 (76%)
History of hyperlipidemia	162 (36%)	66 (29%)	96 (43%)
AKI			
No	152 (34%)	80 (36%)	72 (33%)
Yes	293 (66%)	144 (64%)	149 (67%)

*Median (Q1, Q3); n (%)

## Data Access and Responsibility

Helena Furberg had full access to all the data in the study and takes responsibility for the integrity of the data and the accuracy of the data analysis.

## Data Sharing Policy

Data are available for bona fide researchers who request them from the authors. For more information, contact the senior author (H. Furberg).
